# Remarks on Regularization by Noise, Convex Integration and Spontaneous Stochasticity

**DOI:** 10.1007/s00032-024-00406-8

**Published:** 2024-09-02

**Authors:** Franco Flandoli, Marco Rehmeier

**Affiliations:** 1 Faculty of Sciences, Scuola Normale Superiore, Pisa, Italy; 2 Faculty of Mathematics, Bielefeld University, Bielefeld, Germany

**Keywords:** Convex integration, Regularization by noise, Spontaneous stochasticity, Fluid dynamics, Multiscale systems, 76D05, 35Q31, 60H50, 70K55, 35R60

## Abstract

This note is devoted to a discussion of the potential links and differences between three topics: regularization by noise, convex integration, spontaneous stochasticity. All of them deal with the effect on large scales of a small-scale perturbation of fluid dynamic equations. The effects sometimes have something in common, like convex integration and spontaneous stochasticity, sometimes they look the opposite, as in regularization by noise. We are not aware of rigorous links or precise explanations of the differences, and hope to drive new research with this comparative examination.

## Introduction

Fluids are multiscale systems. To simplify the discussion, assume that a fluid is composed of only two main scales or groups of scales, called below large and small scales. Usually we are interested in the large scales, those which contain most of the energy, those that are visible, that produce the main effects like the drag exerted on solid bodies. Moreover, we tend to think that the large scales produce also small scales by instabilities and drive their behavior; all true facts in general. In these notes we want to explore the opposite direction, namely *the effects that small scales may have on large ones*. We focus on three effects.

The first main effect was identified a long time ago: Joseph Boussinesq wrote, in 1876, that turbulent small scales may have a dissipation effect on large scales. This is the paradigm of Large Eddy Simulations, very useful to reduce the degrees of freedom in numerical simulations, and it is a recognized phenomenon in many real fluids, like channel flows. Recently, a mathematical theory has been constructed which captures some features of this phenomenon and, in particular, it has been used to prove *regularization by noise* results, precisely the delay of blow-up due to noise. We shall review one result of this theory in Sect. 3.

The second one is, somewhat, the opposite. Anisotropic small scale motions may trigger large scale ones and provoke the emergence of large scale structures and organization. Zonal flows in the atmosphere of planets or in Plasma toroidal systems and also the dynamo effect seem to be examples.^1^ We shall not treat these specific physical phenomena, but a mathematical one which is different, but shares with them the large scale impact of small scale oscillations: *convex integration*. It is briefly reviewed in Sect. 4.

The third one concerns the propagation of randomness from very small to large scales. Already Landau and Lifschitz [17] wrote Navier–Stokes equations forced by molecular noise, conjecturing that molecular motion may trigger some effect at Kolmogorov scale and then propagate upward to large scales. Recently, a multiscale dynamical system has been constructed [19] which mimics this cascade effect and shows that *spontaneous stochasticity* for an infinite dimensional system with energy transfer between scales is possible. It is not a result for the Navier–Stokes equations yet, hence we shall not review it. However, we produce an example of a stochastic solution for the Euler equations by means of a convex integration scheme, see Sect. 4. It is still far from being intrinsic as the spontaneous stochastic solutions of [19] but it helps to raise interesting questions.

We dream that a coherent picture of

- Regularization by noise
- Convex integration
- Spontaneous stochasticity

exists. This note does not solve this issue but only poses the problem. Regularization by noise and convex integration are opposite phenomena but some building blocks are similar and the question then is what makes such a basic difference. Convex integration and spontaneous stochasticity may have a lot in common. Recently, in [16], stochasticity, in a more general sense, has already been used in convex integration constructions. We believe that such considerations deserve future investigation. The presence of stochasticity is, moreover, the key for regularization by noise, hence a difficult question is how these features could coexist in the same fluid dynamic model.

This work has been the result of discussions about a difficult open problem, related to a series of talks given by the first author on the occasion of the Riemann Prize 2023 at the Riemann International School of Mathematics. The style of this note is thus a review of known facts considered from a particular perspective, emphasizing questions more than new results (except for a new example in convex integration theory).

## Small Scales Acting on Larger Ones

### Two Approaches

A common element of the three topics outlined in the introduction (regularization by noise, convex integration and spontaneous stochasticity) is the *action of small scales on larger ones*, sometimes similar, sometimes different. This section has the purpose to explain that there are at least two ways to introduce such action, similar but mathematically not equivalent (and maybe with different physical consequences).

The first one, somehow more classical, is through a Reynolds stress tensor, inspired by the decomposition of the solution *u* of Euler or Navier–Stokes equations (here always incompressible and Newtonian)

2.1 $$\begin{aligned} \partial _{t}u+u\cdot \nabla u+\nabla p-\nu \Delta u&=0\nonumber \\ {\text {div}}u&=0 \end{aligned}$$

into two components $u=\overline{u}+u^{\prime }$, one large scale $\overline{u}$ and the other small scale $u^{\prime }$. Assume that the notation $\overline{u}$ stands for $\Lambda u$, where $\Lambda$ is a linear bounded operator in suitable spaces, acting only on the space variable (hence commuting with time derivatives). Then, writing the equation for the large scale $\overline{u}$, under the assumption that $\Lambda$ commutes with spatial derivatives and that boundary conditions do not matter (e.g. because the equation is posed on a torus or full space), we get the equation

$$\begin{aligned} \partial _{t}\overline{u}+\overline{u}\cdot \nabla \overline{u}+\nabla \overline{p}-\nu \Delta \overline{u}&=-{\text {div}}R\left( \overline{u},u^{\prime }\right) \\ {\text {div}}\overline{u}&=0 \end{aligned}$$

(possibly with $\nu =0$) with $\overline{p}=\Lambda p$ (we also assume that $\Lambda$ applies componentwise to vector fields and scalars in the same way). Here, the Reynolds stress tensor $R=R\left( \overline{u},u^{\prime }\right)$ is given by

2.2 $$\begin{aligned} R\left( \overline{u},u^{\prime }\right) =\overline{\left( \overline{u}+u^{\prime }\right) \otimes \left( \overline{u}+u^{\prime }\right) }-\overline{u}\otimes \overline{u} \end{aligned}$$

(which simplifies to $R\left( u^{\prime }\right) =$ $\overline{u^{\prime }\otimes u^{\prime }}$ only in the case of particular operators $\Lambda$, like a global average). Here and throughout, the divergence of a matrix *R*, $\mathop {\textrm{div}}\limits R$, is understood column-wise. So, $R\left( \overline{u},u^{\prime }\right)$ depends on the small scales $u^{\prime }$ (and in general also on $\overline{u}$), but in a rather complex way. This approach is, from the viewpoint of continuum mechanics, more rigorous than the second one outlined below, but suffers the complexity of the form of $R\left( \overline{u},u^{\prime }\right)$.

The second one is inspired by the Lagrangian formulation and better expressed in terms of vorticity. The full analysis is limited to simplified geometries like the full space or a torus because of a lack of boundary conditions for the vorticity. Let us introduce the vorticity $\omega ={\text {curl}}u$ and write the equation for $\omega$:

$$\begin{aligned} \partial _{t}\omega +u\cdot \nabla \omega -\omega \cdot \nabla u-\nu \Delta \omega&=0\\ \omega&={\text {curl}}u\\ {\text {div}}u&=0 \end{aligned}$$

In this case we do not decompose $\omega =\overline{\omega }+\omega ^{\prime }$ in large and small scales but we proceed differently. Inspired by the point vortex method and vortex-wave variants [21, 22], we idealize the fluid as composed of vortex structures

$$\begin{aligned} \omega =\overline{\omega }+\sum _{k\in K}\omega _{k}^{\prime } \end{aligned}$$

(*K* a finite set for simplicity), where $\overline{\omega }$ is large scale and $\left( \omega _{k}^{\prime }\right) _{k\in K}$ are several small scale structures (e.g. almost point vortices in 2D and vortex filaments in 3D). If they have disjoint supports, the evolution of each one is driven by the velocity fields of all the others, including its own (except for point vortices in 2D), namely each vortex structure acts on each other by transport and stretching. Heuristically we apply the same rule in the case of superposition of supports (although the rigorous results are only in the first case). Therefore

$$\begin{aligned} \partial _{t}\overline{\omega }+\overline{u}\cdot \nabla \overline{\omega }-\overline{\omega }\cdot \nabla \overline{u}-\nu \Delta \overline{\omega }&=-\sum _{k\in K}\left( u_{k}^{\prime }\cdot \nabla \overline{\omega } -\overline{\omega }\cdot \nabla u_{k}^{\prime }\right) \\ \overline{\omega }&={\text {curl}}\overline{u}\\ {\text {div}}\overline{u}&=0, \end{aligned}$$

where $u_{k}^{\prime }$ are the velocity fields associated to the small scale structures, namely

$$\begin{aligned} {\text {curl}}u_{k}^{\prime }=\omega _{k}^{\prime },\quad {\text {div}}u_{k}^{\prime }=0. \end{aligned}$$

In a sense, compared to the Reynolds approach above, here the analog of $R\left( \overline{u},u^{\prime }\right)$ is

$$\begin{aligned} L\left( \overline{\omega },\left( u_{k}^{\prime }\right) _{k\in K}\right) :=\sum _{k\in K}\left( u_{k}^{\prime }\cdot \nabla \overline{\omega } -\overline{\omega }\cdot \nabla u_{k}^{\prime }\right) . \end{aligned}$$

The form of this operator is much more explicit than $R\left( \overline{u},u^{\prime }\right)$, it represents, as already said above, the action by transport and stretching of each small vortex on the large scales. The drawback of this approach is the meaning of $\overline{\omega }$ and $u_{k}^{\prime }$: at time zero we may prescribe a subdivision $\omega \left( 0\right) =\overline{\omega }\left( 0\right) +\sum _{k\in K}\omega _{k} ^{\prime }\left( 0\right)$ into large and small scale structures, but during the time evolution the decomposition $\omega \left( t\right) =\overline{\omega }\left( t\right) +\sum _{k\in K}\omega _{k}^{\prime }\left( t\right)$ does not necessarily represent large and small structures anymore, since $\overline{\omega }\left( t\right)$ may develop small structures inside itself and $\omega _{k}^{\prime }\left( t\right)$ may gather together into larger structures (typically occurring in 2D). Let us insist that here we do not have $\overline{\omega }\left( t\right) =\Lambda \omega \left( t\right)$.

One can write a link between the two formulations and see that they differ by a commutator, which however is small only in the limit when the perturbation $u^{\prime }$ becomes negligible, hence it is of moderate importance in the attempt to claim that the two approaches are equivalent. Thus, at present, we do not have precise results which state that the two approaches should be approximately equivalent, although heuristically they describe similar ideas about small scales acting on large ones.

### Deterministic and Stochastic Parametrization and Link with Convex Integration and Regularization by Noise

The operators $R\left( \overline{u},u^{\prime }\right)$ and $L\left( \overline{\omega },\left( u_{k}^{\prime }\right) _{k\in K}\right)$ are not only complex but also depending on $u^{\prime }$ and $u_{k}^{\prime }$ which are not given, but should be the components of a vector, together with $\overline{u}$ and $\overline{\omega }$, a solution of a complex system. In this way we have not reduced the complexity of the model by the introduction of large and small scales.

The only strategy then is introducing simplified models of $u^{\prime }$ and $u_{k}^{\prime }$, which can be done in many different ways and in particular in a deterministic and a stochastic fashion. We call this “parametrization” of $u^{\prime }$ and $u_{k}^{\prime }$.

To keep the exposition to a minimum of variants, let us say that the typical attitude of convex integration theory is replacing $R\left( \overline{u},u^{\prime }\right)$ by a deterministic tensor *R*, which is part of the solution of the problem, or an input, depending on the viewpoint, but no more of the specific form (2.2). The equations take the form (we drop here the bar over the large scales, since only they remain and their link with previous definitions like $\overline{u}=\Lambda u$ disappears)

$$\begin{aligned} \partial _{t}u+u\cdot \nabla u+\nabla p-\nu \Delta u&=-{\text {div}}R\\ {\text {div}}u&=0. \end{aligned}$$

As already said, the main new viewpoint is that $\left( u,p,R\right)$ is a solution (the other is that *R* is given and $\left( u,p\right)$ is a solution). It is a particular form of deterministic parametrization. The final aim is removing *R*, going back to solutions of the true Euler or Navier–Stokes equations. Even if *R*, at each step of the convex integration construction outlined in Sect. 4, incorporates all scales up to a certain one, the smaller scales in such a range are the most important ones in *R*. Thus *R* may be interpreted as a small scale input. The corresponding solution *u* is built in such a way to have a large-scale nontrivial part (for instance values far from zero at time *T* even if $u\left( 0\right) =0$). Thus $\left( u,p,R\right)$ is constructed in such a way to have small scale input *R* and large scale output *u*.

On the other hand, the typical attitude of regularization by noise is replacing $L\left( \overline{\omega },\left( u_{k}^{\prime }\right) _{k\in K}\right)$ by a stochastic term, usually of white noise type, Stratonovich sense (to preserve invariants and to satisfy heuristically the Wong–Zakai principle, see other expositions on this issue, like [13]). In principle, the form of the replacement should be

$$\begin{aligned} L\left( \overline{\omega },\left( u_{k}^{\prime }\right) _{k\in K}\right) \rightarrow \sum _{k\in K}\left( \sigma _{k}\cdot \nabla \omega -\omega \cdot \nabla \sigma _{k}\right) \circ \frac{dW_{t}^{k}}{dt}, \end{aligned}$$

where $\sigma _{k}=\sigma _{k}\left( x\right)$ are smooth divergence free fields and $W_{t}^{k}$ are independent Brownian motions. However, this problem proved to be too difficult because of the stochastic stretching term, and thus the only results proved until now are related to the simplified replacement

$$\begin{aligned} L\left( \overline{\omega },\left( u_{k}^{\prime }\right) _{k\in K}\right) \rightarrow \sum _{k\in K}\Pi \left( \sigma _{k}\cdot \nabla \omega \right) \circ \frac{dW_{t}^{k}}{dt}, \end{aligned}$$

where the details of the projection $\Pi$ are given at the beginning of Sect. 3. Including $\Pi$ is necessary since the sum of all other terms of the equation is divergence free, hence also the additional must be, in order that solutions could exist. This is a stochastic parameterization. The equation becomes an SPDE, of the form

2.3 $$\begin{aligned} d\omega +\left( u\cdot \nabla \omega -\omega \cdot \nabla u-\nu \Delta \omega \right) dt=-\sum _{k\in K}\Pi \left( \sigma _{k}\cdot \nabla \omega \right) \circ dW_{t}^{k}. \end{aligned}$$

Since $dW_{t}^{k}$ is fastly varying in time and the choice of $\sigma _{k}$ made below is of small scale structures, also this model has the form of small scales (randomly) acting on large ones. The final result is opposite to the one of convex integration: the effect of the small scales is to smooth the potential blow-up of $\omega$, to reduce its intensity. The first reason to write this note was to emphasize this difference in behavior between the deterministic parametrization of Reynolds type and the stochastic parametrization of Lagrangian type.

### About a Notion of Spontaneous Stochasticity

The concept of spontaneous stochasticity is not unique and a definition may depend on the framework; for instance the examples of stochastic behavior for Peano phenomena proved in [1] are examples of spontaneous stochasticity, but disjoint from the fluid equations considered here. In fluid dynamics, spontaneous stochasticity may be interpreted in a Lagrangian framework (see for instance [23]) or in the Eulerian formulation, now discussed. Following [2] and related works quoted therein, with ideas going back to [17], we ask ourselves here whether it is possible to identify a definition.

A reasonable heuristic idea for fluids (specific of fluids, where the interaction of scales is strong and we have specific intuitions about relative influence of scales) could be the following one. We perturb the Euler or Navier–Stokes equations by stochastic small scales, we observe the effect produced on large scales and we send the small scale perturbation to zero. If the effect on large scales is maintained in the limit, namely the limit is still truly stochastic, and the limit effect is sufficiently universal with respect to details of the small scale perturbations, we say that we observe spontaneous stochasticity.

Several choices should be made in order to formulate rigorously this heuristic idea:

- we have to choose a class of admissible solutions (in particular because convex integration solutions and classical Leray weak solutions still remain potentially disjoint classes);
- we have to choose a class of random perturbations (e.g. white noise in time or smooth in time, Gaussian or more general from the statistical viewpoint, or more specific like bounded noise; this detail may be important for convex integration, since the Reynolds stresses there should satisfy certain bounds);
- we have to choose the notion of convergence to zero of the stochastic perturbation (e.g. convergence in a classical norm like the uniform one, or convergence against test functions, more suitable to account for smaller and smaller scales which are not infinitesimal in classical norms);
- we have to choose either the Reynolds formulation or the Lagrangian one, see the previous subsections, to impose the action of small scales on large ones.

Due to the difficulty to make a choice in absence of results (the topic is still open), we prefer to first give a definition of stochastic solution of the deterministic Euler or Navier–Stokes equations, a definition where the number of choices is less wide. Then we give a heuristic definition of spontaneous stochasticity.

First, let us introduce a very general class of solutions which may accommodate various subclasses. We choose the Reynolds formulation. Consider the Euler ($\nu =0$) or Navier–Stokes ($\nu >0$) equations on the torus $\mathbb {T}^{d}$ and on $\left[ 0,T\right]$:

$$\begin{aligned} \partial _{t}u+u\cdot \nabla u+\nabla p-\nu \Delta u&={\text {div}}R\\ {\text {div}}u&=0\\ u|_{t=0}&=0 \end{aligned}$$

(we choose the zero initial condition to avoid unnecessary general definitions). Call $\mathbb {R}_{sym}^{d\times d}$ the set of all symmetric $d\times d$ matrices. We may impose on them the property of zero trace, putting the trace into the term $\nabla p$. Denote by $L_{\sigma }^{2}\left( \left[ 0,T\right] \times \mathbb {T}^{d};\mathbb {R}^{d}\right)$ the space of solenoidal (in the distributional sense) and mean zero vector fields $u\in L^{2}\left( \left[ 0,T\right] \times \mathbb {T}^{d};\mathbb {R}^{d}\right)$; similarly for $C^{1}\left( \left[ 0,T\right] ;C_{\sigma }^{2}\left( \mathbb {T}^{d};\mathbb {R}^{d}\right) \right)$.

#### Definition 2.1

Assume $R\in L^{1}\left( \left[ 0,T\right] \times \mathbb {T}^{d} ;\mathbb {R}_{sym}^{d\times d}\right)$. Call very weak solution any vector field $u\in L_{\sigma }^{2}\left( \left[ 0,T\right] \times \mathbb {T} ^{d};\mathbb {R}^{d}\right)$ such that

$$\begin{aligned} \int _{0}^{T}\int _{\mathbb {T}^{d}}\left( u\cdot \partial _{t}\phi +Trace\left( \left( u\otimes u-R\right) \nabla \phi \right) +\nu u\cdot \Delta \phi \right) dxdt=0 \end{aligned}$$

for all test vector fields $\phi \in C^{1}\left( \left[ 0,T\right] ;C_{\sigma }^{2}\left( \mathbb {T}^{d};\mathbb {R}^{d}\right) \right)$ such that $\phi \left( T\right) =0$. Call $\mathcal {S}_{R}$ the set of all very weak solutions. When $R=0$, we denote by $\mathcal {S}$ the set $\mathcal {S}_{R}$.

If *R* is a random variable, defined on a probability space $\left( \Omega ,F,\mathbb {P}\right)$, with values in $L^{1}\left( \left[ 0,T\right] \times \mathbb {T}^{d};\mathbb {R}_{sym}^{d\times d}\right)$, and *u* is a random variable on $\left( \Omega ,F,\mathbb {P}\right)$, such that $u\left( \omega \right)$ is a very weak solution corresponding to $R\left( \omega \right)$, for $\mathbb {P}$-a.e. $\omega \in \Omega$, then we call *u* a random very weak solution corresponding to *R*.

#### Definition 2.2

Given a sequence $\mathcal {R}=\left( R_{n}\right) _{n\in \mathbb {N}}$ of random variables with values in $L^{1}\left( \left[ 0,T\right] \times \mathbb {T}^{d};\mathbb {R}_{sym}^{d\times d}\right)$, defined on a probability space $\left( \Omega ,F,\mathbb {P}\right)$, we say that there is a nontrivial stochastic solution of the equation

$$\begin{aligned} \partial _{t}u+u\cdot \nabla u+\nabla p-\nu \Delta u&=0\\ {\text {div}}u&=0\\ u|_{t=0}&=0 \end{aligned}$$

corresponding to the sequence $\mathcal {R}$ if there is a sequence of random very weak solutions $\left( u_{n}\right) _{n\in \mathbb {N}}$, $u_{n}$ corresponding to $R_{n}$ for every $n\in \mathbb {N}$, such that the distributions of $u_{n}$ weakly converge to a Borel probability measure *P* on $L_{\sigma }^{2}\left( \left[ 0,T\right] \times \mathbb {T}^{d};\mathbb {R}^{d}\right)$ such that $P\left( \mathcal {S}\right) =1$^2^ and the support of *P* is not a singleton (*P* will be called nontrivial stochastic solution).

If in the setting of the previous definition the sequence of stochastic solutions $(u_n)_{n\in \mathbb {N}}$ converges pathwise to a process *u* in a suitable sense, then a natural candidate for *P* is $\mathbb {P}_u$, the distribution of *u*. However, we stress that such a pathwise convergence is not assumed in general.

#### Remark 2.3

We could give a definition of nontrivial stochastic solution not related to any sequence, just by postulating $P\left( \mathcal {S}\right) =1$ and the support of *P* not being a singleton. However, the existence of such *P* is equivalent to non-uniqueness, namely that $\mathcal {S}$ is not a singleton. Therefore, it would be a poor definition.

#### Remark 2.4

The definition above, corresponding to a sequence $\mathcal {R}$, is just a bit deeper. A theorem of existence of a stochastic solution corresponding to a sequence $\mathcal {R}$ would be very deep if we may prescribe $\mathcal {R}$ a priori on a physical ground. Unfortunately, at present we may only construct both $\mathcal {R}$ and a corresponding sequence of random very weak solutions $\left( u_{n}\right) _{n\in \mathbb {N}}$, and their limit in law *P*.

#### Remark 2.5

Even if not explicitly required (as in Definition 2.2, to avoid a decision about the topology of convergence) that $R_{n}$ goes to zero, it is clear that, in order to prove that the limit law *P* is concentrated on $\mathcal {S}$ it is necessary that $R_{n}$ goes to zero, at least in some suitable weak sense.

We give an example of a nontrivial stochastic solution to the 3*D* Euler equations in Sect. 4.4.

From nontrivial stochastic solutions to spontaneous stochasticity. Based on the previous precise definition, we could now say more heuristically that *spontaneous stochasticity holds *if there exists a nontrivial stochastic solution *P* corresponding (simultaneously) to a large family of natural sequences $\mathcal {R}$. Clearly the notions of “large family” and “natural sequences” should be quantified rigorously and this is not appropriate to be done without any idea of a theorem which could be proved, or at least a closer investigation of the random perturbations that are “natural” from the physical viewpoint. Moreover, convergence of the full sequence $(\mathbb {P}_{u_n})_{n\in \mathbb {N}}$ might be too much of a requirement, hence we may relax the notion of spontaneous stochasticity to the existence of a (possibly non-unique) non-Dirac weak limit point $P'$ of $(\mathbb {P}_{u_n})_{n\in \mathbb {N}}$ with $P'(\mathcal {S}) = 1$.

At present there are no results of spontaneous stochasticity of this form, or even much weaker. But the results for conceptual models like [19] go in this direction. Also, following this work, a promising approach could be looking for nontrivial stochastic solutions related to fixed points of renormalization group iterations. Maybe convex integration schemes can be recasted as renormalization group iterations.

### Models of Small Scales

Although the literature is rich of variants, we could simplify and say that essentially in both regularization by noise and convex integration, there are two classes of small-scale perturbations:

- Fourier-type oscillations
- spatially-localized fluid structures.

The noise used in studies of regularization by noise is either expressed as a Fourier series, where the relevant contribution comes from high frequencies with low intensity, or it is based on vortex structures, of small size and intensity and high cardinality and good degree of space-covering. The two categories of perturbations mostly used in convex integration are Beltrami flows, which are suitably refined Fourier components, or Mikado flows, which are compact support velocity structures. Maybe this similarity between the two theories is only superficial, but it must be remarked.

A technical difference is that classical vortex structures in 2D are invariant by rotation and, as such, would be useless in convex integration. Indeed, a key step in convex integration construction is that small scale perturbations “span all directions” in a suitable sense. Both Beltrami flow and Mikado flows have a direction, and by using suitable collections of them, one can “span” the necessary directions. Invariant by rotation vortex structures do not have this richness. Maybe the breaking of symmetry due to the directions of convex integration structures is a deep element for the production of organized results, opposite to the dispersion of information behind regularization by noise.

## Review of a Regularization by Noise Result

The theory of regularization by noise (which now has even a title in the Mathematical Classification) is wide, with many directions; a partial overview can be found in [10]. Here we concentrate only on one of the recent developments, initiated in [11], and specifically describe the result of [12].

Consider, on $\mathbb {T}^{3}$, the vorticity equation (2.3) with $\nu >0$, where $\mathop {\textrm{curl}}\limits u=\omega$, $\mathop {\textrm{div}}\limits u=0$ and an initial condition $\omega |_{t=0}=\omega _{0}$ is specified. Consider the space of square integrable vector fields, $L^{2}\left( \mathbb {T}^{3}; \mathbb {R}^{3}\right)$, with the classical $L^{2}$ norm and denote by *H* the closure in $L^{2}\left( \mathbb {T}^{3};\mathbb {R}^{3}\right)$ of the set of divergence free, mean zero, smooth vector fields from $\mathbb {T}^{3}$ to $\mathbb {R}^{3}$ (hence periodic). Taken $v\in L^{2}\left( \mathbb {T}^{3}; \mathbb {R}^{3}\right)$, mean zero, there is a unique $w\in H$ and a unique (up to a constant) $q\in H^{1}\left( \mathbb {T}^{3};\mathbb {R}\right)$ such that $v=w+\nabla q$ (Helmholtz decomposition). We call *w* the projection of $L^{2}\left( \mathbb {T}^{3};\mathbb {R}^{3}\right)$ on *H* and call $\Pi$ the projection operator, $w=\Pi v$ (the operator already used in Sect. 2.2). The noise, as already remarked, is not fully motivated on a physical ground (see the discussion in [12]), but it may be a first step in the direction of a more complete result. By solution $\omega$ we mean a continuous adapted process in *H*, with $\omega |_{t=0}=\omega _{0}$, satisfying Eq. (2.3) written in integral Itô form, weakly against test functions, after the (formal) Stratonovich integral has been converted into an Itô integral plus the Itô-Stratonovich corrector. Since these details do not add so much to the present discussion, we address the reader to [12] for them.

### Theorem 3.1

Given $c_{0},\varepsilon >0$ there exists a noise $\sum _{k\in K}\sigma _{k}\left( x\right) W_{t}^{k}$, with the following property: for every initial condition $\omega _{0}\in H$ with $\left\| \omega _{0}\right\| _{L^{2}}\le c_{0}$, the stochastic Navier–Stokes equations (2.3) have a global unique solution, up to probability $\varepsilon$. Namely, the maximal time $\tau$ of existence and uniqueness in *H* satisfies

$$\begin{aligned} \mathbb {P}\left( \tau <\infty \right) \le \varepsilon . \end{aligned}$$

In order to appreciate the strength of this result, let us recall what is known in the deterministic case: only that given $\omega _{0}\in H$, there exists a unique *maximal* solution

$$\begin{aligned} \omega \in C\left( [0,\tau );H\right) . \end{aligned}$$

If $\left\| \omega _{0}\right\| _{L^{2}}$ is small enough, the solution is global, $\tau =+\infty$, the smallness of $\left\| \omega _{0}\right\| _{L^{2}}$ depending on the viscosity $\nu$. The open problem is whether this solution blows up:

$$\begin{aligned} \tau <\infty \text {, }\lim _{t\uparrow \tau }\left\| \omega \left( t\right) \right\| _{L^{2}}=+\infty . \end{aligned}$$

The result above shows that transport noise improves the control of $\left\| \omega \left( t\right) \right\| _{L^{2}}$ and prevents blow-up, up to a small probability event.

To understand the technical reason, recall that in the deterministic case, by simple energy type estimates, the norm $\left\| \omega \left( t\right) \right\| _{L^{2}}^{2}$ can be controlled *locally*:

$$\begin{aligned} \frac{1}{2}\frac{d}{dt}\left\| \omega \left( t\right) \right\| _{L^{2} }^{2}+\nu \left\| \nabla \omega \left( t\right) \right\| _{L^{2}} ^{2}=\left\langle \omega \cdot \nabla u,\omega \right\rangle _{L^{2}}. \end{aligned}$$

The term $\left\langle \omega \cdot \nabla u,\omega \right\rangle _{L^{2}}$ describes the *stretching* of vorticity $\omega$ produced by the deformation tensor $\nabla u$. This is the potential source of unboundedness of $\left\| \omega \left( t\right) \right\| _{L^{2}}^{2}$. Sobolev and interpolation inequalities give us:

$$\begin{aligned} \left\langle \omega \cdot \nabla u,\omega \right\rangle _{L^{2}}&\le \left\| \omega \right\| _{L^{3}}^{3}\le \left\| \omega \right\| _{W^{1/2,2}}^{3}\le \left\| \omega \right\| _{L^{2}}^{3/2}\left\| \omega \right\| _{W^{1,2}}^{3/2}\\&\le \nu \left\| \omega \right\| _{W^{1,2}}^{2}+\frac{C}{\nu ^{3} }\left\| \omega \right\| _{L^{2}}^{6},\end{aligned}$$

which leads to

$$\begin{aligned} \frac{d}{dt}\left\| \omega \left( t\right) \right\| _{L^{2}}^{2} \le \frac{C}{\nu ^{3}}\left\| \omega \right\| _{L^{2}}^{6}. \end{aligned}$$

From this inequality we may only deduce a local control on $\left\| \omega \left( t\right) \right\| _{L^{2}}^{2}$ unless $\left\| \omega _{0}\right\| _{L^{2}}^{2}$ is so small that the larger power on the right-hand-side provides an improvement of the estimate instead of a deterioration. The interval of existence given by the previous inequality depends on the viscosity coefficient $\nu$. The (potential) explosion is delayed for large $\nu$; but in real fluids it is a very small constant, for instance of order of $10^{-5}$.

The key for a regularization by noise stands in the fact that transport noise improves dissipation, increases the viscosity constant by a term called eddy viscosity in turbulence theory; hence it delays blow-up. One way to get an intuition of the reason (for the rigorous proof see [12]) is to mention the following scaling limit theorem.

### Theorem 3.2

Let $\omega _{0}\in H$ and $\left[ 0,T\right]$ be given. Let $\sum _{k\in K}\sigma _{k}^{N}\left( x\right) W_{t}^{k}$ be the sequence of noises defined below, with the intensity constant $\nu _T$. Then the corresponding solutions $\omega ^N$ converge in probability to the solution of

$$\begin{aligned} \partial _{t}\omega +u\cdot \nabla \omega - \omega \cdot \nabla u=\left( \nu +\frac{3}{5}\nu _{T}\right) \Delta \omega \end{aligned}$$

in the topology of $C\left( \left[ 0,T\right] ;H^{-\delta }\right)$. It follows that for large *N* the norm $\left\| \omega ^{N}\left( t\right) \right\| _{H^{-\delta }}^{2}$ is bounded on $\left[ 0,T\right]$, with high probability (implying well-posedness of $\omega ^{N}$).

For the specific purpose of this paper, where we want to compare small scale inputs and their effect, we limit ourselves to a short description of the noise used in these results.

Decompose $\mathbb {Z}_{0}^{3} = \mathbb {Z}^3\backslash \{0\}$ as $\mathbb {Z}_{+}^{3}\cup \left( -\mathbb {Z}_{+}^{3}\right)$. Call

$$\begin{aligned} e_{k,\alpha }\left( x\right)&=a_{k,\alpha }\cos \left( 2\pi k\cdot x\right) \\ e_{-k,\alpha }\left( x\right)&=a_{k,\alpha }\sin \left( 2\pi k\cdot x\right) \end{aligned}$$

for $k\in \mathbb {Z}_{+}^{3},\alpha =1,2$, with $\left\{ a_{k,1},a_{k,2} ,\frac{k}{\left| k\right| }\right\}$ being an orthonormal basis of $\mathbb {R}^{3}$. This family of vector fields is a complete orthonormal system of *H*. Set

$$\begin{aligned} \sigma _{k,\alpha }\left( x\right) =\theta _{k}e_{k,\alpha }\left( x\right) \end{aligned}$$

with $\theta$ radially symmetric: $\theta _{k}=\theta _{h}$ if $\left| k\right| =\left| h\right|$. One has

$$\begin{aligned} \frac{1}{2}\sum _{k,\alpha }\sigma _{k,\alpha }\left( x\right) \otimes \sigma _{k,\alpha }\left( x\right) \sim \left\| \theta \right\| _{L^{2} }^{2}I. \end{aligned}$$

This property, very vaguely, is at the foundations of the fact that an additional Laplacian comes out from this kind of noise. Given $\nu _{T}>0$, specialize $\theta$, depending on a parameter $N\in \mathbb {N}$:

$$\begin{aligned} D_{N}=\left\{ k\in \mathbb {Z}_{0}^{3}:N\le \left| k\right| \le 2N\right\} ,\qquad \theta _{k}^{N}\sim \sqrt{\nu _{T}}N^{-3/2}1_{D_{N}} \end{aligned}$$

so that $\left\| \theta ^{N}\right\| _{L^{2}}^{2}\sim \nu _{T}$. These are the parameters necessary to get the result of the previous theorem.

Let us notice that the fields used here are very close to the Beltrami flows used below in the convex integration scheme.

## Convex Integration and Spontaneous Stochasticity

In recent years, convex integration led to significant progress concerning the construction and ill-posedness of very weak solutions to the Euler and Navier–Stokes equations. Rooted in Nash’s proof of the $C^1$ isometric embedding problem 70 years ago, after a series of improvements it led to a proof of the flexible part of Onsager’s conjecture [5, 15]: On $\mathbb {T}^3$, for every $0< \beta < \frac{1}{3}$, there is a very weak solution $v\in C^\beta ([0,T]\times \mathbb {T}^3; \mathbb {R}^3)$ to (2.1) ($\nu = 0$) with strictly decreasing kinetic energy, i.e. $\frac{d}{dt}||v(t)||_{L^2} <0$. Actually, in [5] solutions with any smooth strictly positive kinetic energy profile are constructed. A similar result holds for the 3*D* Navier–Stokes equations, albeit its constructed solutions are much less regular [7]. There are excellent review papers on these matters, including discussions of previous and related works, for instance [6, 8]. We limit ourselves here to a very brief repetition of the general scheme, based on Beltrami flows.

### The Convex Integration Scheme

Convex integration solutions to (2.1), $\nu = 0$, are constructed as limits of smooth solutions $(v_q,p_q,R_q)_{q\in \mathbb {N}}$ to the Euler–Reynolds equation

4.1 $$\begin{aligned} \partial _t v_q +( v_q\cdot \nabla ) v_q + \nabla p_q = \mathop {\textrm{div}}\limits R_q,\quad \quad \mathop {\textrm{div}}\limits v_q = 0, \end{aligned}$$

where $R_q\in \mathbb {R}^{3\times 3}_{sym}$. If $R_q \xrightarrow {C^0}0$ and $v_q \xrightarrow {C^\beta }v$, then *v* is a very weak solution to (2.1) in $C^\beta$. The iterative construction of $(v_q,R_q)$ (we omit the pressure, since it does not play an essential role) proceeds via perturbing $v_q$ by a highly oscillating vector field $w_{q+1}$, i.e.

4.2 $$\begin{aligned} v_{q+1}:= v_q + w_{q+1}, \end{aligned}$$

and $R_{q+1}$ is calculated from (4.1) at stage $q+1$. Convergence of $R_q$ and $v_q$ is ensured by estimates

A1 $$\begin{aligned} ||w_{q+1}||_{C^0}&\le \delta _{q}^{\frac{1}{2}} \end{aligned}$$

A2 $$\begin{aligned} ||w_{q+1}||_{C^1}&\le \delta _{q}^{\frac{1}{2}}\lambda _q \end{aligned}$$

A3 $$\begin{aligned} ||R_{q}||_{C^0}&\lesssim \delta _{q+1}, \end{aligned}$$

for suitable sequences $(\delta _q)_{q \in \mathbb {N}_0},(\lambda _q)_{q \in \mathbb {N}_0}$ converging to 0 and $+\infty$, respectively. One can think of $\lambda _q = a^{2^q}$, $\delta _q = \lambda _q^{-c_0}$ for some $c_0>0$ and $a \gg 1$, but actual choices are slightly more involved. By interpolation, (A1)–(A2) yields $v_q \xrightarrow {C^\beta } v$, where $\beta <\frac{1}{3}$ depends on $(\delta _q)_{q \in \mathbb {N}}$ and $(\lambda _q)_{q\in \mathbb {N}}$. The main work is to construct $w_{q+1}$ so that (A1)–(A3) are satisfied for all $q \in \mathbb {N}_0$. By an additional iterative estimate, *v* can attain any prescribed smooth strictly positive kinetic energy profile.

Instead of prescribing energies, here we concern ourselves with solutions with zero initial condition, following [3] (see also [4, 14, 15]), where solutions with compactly supported kinetic energy profiles (which cannot be prescribed) in (0, *T*) were constructed. The reason we pursue this direction is explained in Remark 4.1. With minor changes, this construction produces solutions with energies *e* such that $e = 0$ on $[0,t_0]$ for some $t_0>0$ and $\mathop {\textrm{supp}}\limits e = [t_0,T]$. Since our main concern is not the optimal regularity of solutions, we do not focus on the additional estimates required in [3] for the spatial $C^{\frac{1}{3} -}$-regularity of solutions, but instead follow the simpler construction of $v \in C^{\beta }([0,T]\times \mathbb {T}^3; \mathbb {R}^3)$, $0<\beta < \frac{1}{5}$.

### A Reinterpretation of the Iteration

Here we reinterpret the convex integration scheme of [3] in an abstract and simplified manner. For interested readers, more details regarding the definition of $F_q$ and $G_q$ are presented in the appendix.

Let $(v_q,R_q)$ be a solution to (4.1). We aim to express the construction of $(v_{q+1},R_{q+1})$ as

4.3 $$\begin{aligned}  &   v_{q+1}= v_q + F_q(v_q,R_q) \end{aligned}$$

4.4 $$\begin{aligned}  &   R_{q+1} = G_q\big (v_q,R_q,F_q(v_q,R_q)\big ) \end{aligned}$$

for maps $F_q: (v,R)\mapsto F_q(v,R)$ and $G_q:(v,R,w)\mapsto G_q(v,R,w)$, described now. Comparing with (4.2), we have $F_q(v_q,R_q) = w_{q+1}$. For $(v,R)\in C^1([0,T]\times \mathbb {T}^3;\mathbb {R}^3)\times C^1([0,T]\times \mathbb {T}^3;\mathbb {R}^{3\times 3}_{sym})$, the principal ansatz for the definition of $F_q$ is

4.5 $$\begin{aligned} F_q(v,R) \approx \sum _k a^k_q(v,R)W^k_q, \end{aligned}$$

where $a_q^k(v,R): [0,T]\times \mathbb {T}^3 \rightarrow \mathbb {R}$ are of order $||R||_{C^0}$, and $W^k_q: \mathbb {T}^3\rightarrow \mathbb {R}^3$ are finitely many vector fields of amplitude 1 and frequency $\lambda _{q+1} \gg 1$, called *Beltrami waves*, see Lemma (A.1). The definition of $F_q$ (compare the appendix) is tailored to achieve

4.6 $$\begin{aligned} F_q(v_q,R_q)\otimes F_q(v_q,R_q) +R_q \approx 0. \end{aligned}$$

Regarding $G_q$ we have

4.7 $$\begin{aligned} G_{q}(v,R,w) \approx w \otimes w + R. \end{aligned}$$

We stress that (4.6), (4.7) are oversimplifications. In particular, the LHS of (4.6) is not necessarily close to 0, but consists of high frequency terms, to which an inverse divergence-operator is applied, which renders these terms small. The precise definition of $G_q$ depends on *v* and *q*, even though this is not visible here. By means of $F_q$ and $G_q$, the construction of $R_{q+1}$ is then reformulated as

$$\begin{aligned} G_{q}\big (v_q,R_q,F_{q}(v_q,R_q)\big )= R_{q+1}, \end{aligned}$$

and the iteration (started from a suitable initial pair $(v_0,R_0)$) leading to a very weak solution *v* to (2.1) (recall $\nu = 0$) can thus be restated as4.8Based on this reinterpretation of the iteration scheme, we now pose several questions, relating convex integration to the notion of stochastic solutions and spontaneous stochasticity from Sect. 2.3.

### Questions Regarding the Iteration

Our ultimate goal would be to construct spontaneous stochasticity solutions via a randomized convex integration scheme. In particular, the Reynolds errors $R_q$ appearing in convex integration need to be randomized. Since each of its realizations should still allow for the usual convex integration iteration, this raises technical questions, for instance on the set of matrices which can be allowed as Reynolds errors. The purpose of this section is to present and briefly discuss these questions.

Denote by $D(F_q)$ and $D(G_q)$ the domains of $F_q$,

$$\begin{aligned} F_q: D(F_q)\subseteq C^2([0,T]\times \mathbb {T}^3;\mathbb {R}^3)\times C^2([0,T]\times \mathbb {T}^3;\mathbb {R}^{3\times 3}_{sym}) \rightarrow C^1([0,T]\times \mathbb {T}^3;\mathbb {R}^3) \end{aligned}$$

and $$G_q$$,

$$\begin{aligned} G_q:D(G_q)\subseteq &   C^2([0,T]\times \mathbb {T}^3;\mathbb {R}^3)\times C^2([0,T]\times \mathbb {T}^3;\mathbb {R}^{3\times 3}_{sym})\\  &   \times C^1([0,T]\times \mathbb {T}^3;\mathbb {R}^3)\rightarrow C^1([0,T]\times \mathbb {T}^3;\mathbb {R}^{3\times 3}_{sym}). \end{aligned}$$

Denote by $D_{ER }(F_q)\subseteq D(F_q)$ the subset of pairs (*v*, *R*) solving (4.1) and satisfying the iterative estimates from [3] at stage *q*. Let $D_{F_q}(G_q)\subseteq D(G_q)$ be the set of triples (*v*, *R*, *w*) with $w = F_q(v,R)$, and $D^*_{F_q}(G_q)\subseteq D_{F_q}(G_q)$ those triples with $(v_q,R_q)\in D_{ER}(F_q)$. Throughout (4.8) we have $\big (v_q,R_q,F_q(v_q,R_q)\big )\in D^*_{F_q}(G_q)$ and $\big (v_q+F_q(v_q,R_q),G_q(v_q,R_q,F_q(v_q,R_q))\big ) \in D_{ER }(F_{q+1})$. With the following questions, we intend to initiate a discussion on connections between convex integration, stochastic solutions to the Euler equations, and, eventually, spontaneous stochasticity.

(i) Can the domains $D(F_q)$, $D_{ER }(F_q)$, $D(G_q)$, $D_{F_q}(G_q)$ and $D^*_{F_q}(G_q)$, as well as the range of $F_q$ and $G_q$ on any of these domains be characterized?
(ii) After constructing $(v_0,R_0),\ldots ,(v_q,R_q),(v_{q+1},R_{q+1})$, replace $R_{q+1}$ by a perturbation $\tilde{R}_{q+1} \approx R_{q+1}$ satisfying the same iterative estimate. Is there $(\tilde{v}_q,\tilde{R}_q) \subseteq D_{ER }(F_q)$ with
  $$\begin{aligned}  &   G_q(\tilde{v}_q,\tilde{R}_q,F_q(\tilde{v}_q,\tilde{R}_q))=\tilde{R}_{q+1}\quad \text {and}\quad \big (\tilde{v}_q+F_q(\tilde{v}_q,\tilde{R}_q), \tilde{R}_{q+1}\big ) \in D_{ER}(F_{q+1})? \end{aligned}$$
(iii) Is there a bound (upper or lower) on $|\tilde{v}_q-v_q|_0$ in terms of $\tilde{R}_q-R_q$? We do not expect such a result by general theory of inhomogeneous Euler equations, but maybe the specific convex integration construction of $\tilde{v}_q$ and $v_q$ yields an affirmative answer.
(iv) Alternatively to (ii), are there perturbations $\tilde{F}_q$ and $\tilde{G}_q$ of $F_q$ and $G_q$ as well as $(\tilde{v}_q,\tilde{R}_q)\in D_{ER }(\tilde{F}_q)$ with
  $$\begin{aligned}  &   \tilde{G}_q(\tilde{v}_q,\tilde{R}_q,\tilde{F}_q(\tilde{v}_q,\tilde{R}_q)) = \tilde{R}_{q+1}\quad \text {and}\quad \big (\tilde{v}_q+\tilde{F}_q(\tilde{v}_q,\tilde{R}_q), \tilde{R}_{q+1}\big ) \in D_{ER}(\tilde{F}_{q+1})? \end{aligned}$$
(v) Can (ii) or (iv) be iterated to construct $(\tilde{v}_p,\tilde{R}_p)_{p \ge q}$ such that each pair $(\tilde{v}_p,\tilde{R}_p)$ satisfies the same iterative estimates as $(v_p,R_p)$? If so, this yields limits
  $$\begin{aligned} \tilde{R}_p \xrightarrow {C^0}0,\quad \tilde{v}_p\xrightarrow {C^\beta }\tilde{v}, \end{aligned}$$
  and $\tilde{v}$ is a very weak solution to (2.1). We ask whether $\tilde{v}\ne v$.

The first question seems difficult. For (iv), we have a positive example, see Sect. 4.4. These questions are closely related to stochastic solutions to the Euler equations. Indeed, if $\tilde{R}_{q+1}$ is a random variable on a probability space $(\Omega , F, \mathbb {P})$ with values in the range of $G_q$ on $D^*_{F_q}(G_q)$, then positive answers to (ii), (iv), (v) for the realizations of $\tilde{R}_{q+1}$ would yield that the distributions $\mathbb {P}_{\tilde{v}_q}$ weakly converges to the distribution $\mathbb {P}_{\tilde{v}}$ of the pathwise limit $\tilde{v}$, and $\mathbb {P}_{\tilde{v}}(\mathcal {S}) = 1$ (see Definition 2.2). A natural question is

(vi) How do $\mathbb {P}_{\tilde{R}_{q+1}}$, $\mathbb {P}_{\tilde{v}_{q+1}}$ and $\mathbb {P}_{\tilde{v}}$ compare? In particular, is $\mathbb {P}_{\tilde{v}}$ not a Dirac measure, i.e. a nontrivial stochastic solution? Say, if $\mathbb {P}_{\tilde{R}_{q+1}}$ is Gaussian or uniform in a suitable sense, what can be said about $\mathbb {P}_{\tilde{v}_{q+1}}$ and $\mathbb {P}_{\tilde{v}}$?

In the following subsection we give positive answers to some of these questions via a specific example.

### Example: A Nontrivial Stochastic Solution to 3*D* Euler Equations

Let $q\in \mathbb {N}_0$, $\alpha \in [0,1]$, and set

$$\begin{aligned} \tilde{R}_{q+1}(t,x):= \alpha R_{q+1}(\sqrt{\alpha }t,x), \end{aligned}$$

where $R_{q+1}$ is the Reynolds stress term for (4.1) obtained after $q+1$ iterations of the convex integration scheme. There are maps $\tilde{F}_q,\tilde{G}_q$ and a pair $(\tilde{v}_q,\tilde{R}_q) \in D_{ER }(\tilde{F}_q)$ such that $\tilde{G}_q\big (\tilde{v}_q,\tilde{R}_q,\tilde{F}_q(\tilde{v}_q,\tilde{R}_q) \big ) = \tilde{R}_{q+1}$. Indeed, define

$$\begin{aligned} \tilde{v}_{q}(t,x):= \sqrt{\alpha }v_q(\sqrt{\alpha }t, x),\quad \tilde{R}_q(t,x):= \alpha R_q(\sqrt{\alpha }t, x), \quad (t,x)\in [0,T]\times \mathbb {T}^3. \end{aligned}$$

$(\tilde{v}_q,\tilde{R}_q)$ solves (4.1) pointwise and satisfies the same iterative estimates as $(v_q,R_q)$. Defining $\tilde{F}_q$ analogously to $F_q$, but with $\tilde{\mu }_q:= \sqrt{\alpha }\mu _q$ instead of $\mu _q$ (see the appendix), we obtain $(\tilde{v}_q,\tilde{R}_q) \in D_{ER}(\tilde{F}_q)$, and

4.9 $$\begin{aligned} \tilde{F}_q(\tilde{v}_q,\tilde{R}_q)(t,x) = \sqrt{\alpha }F_q(v_q,R_q)(\sqrt{\alpha }t,x). \end{aligned}$$

Indeed, to see this it suffices to compare with the appendix in order to note

4.10 $$\begin{aligned} \tilde{R}^l_q(t,x) = \alpha R^l_q(\sqrt{\alpha }t,x) \end{aligned}$$

and

$$\begin{aligned} \tilde{\Phi }^l_q(t,x) = \Phi ^l_q(\sqrt{\alpha }t,x), \end{aligned}$$

where the left-hand sides are defined as in (A.1) and (A.2), with $(\tilde{v}_q,\tilde{R}_q)$ in place of (*v*, *R*), and $\tilde{\mu }_q$ in place of $\mu _q$. Similarly, define $\tilde{G}_{q}$ as $G_q$, but with $\tilde{\mu }_q$ instead of $\mu _q$. By (A.3), (4.9), (4.10) it follows

$$\begin{aligned} \tilde{G}_q(\tilde{v}_q,\tilde{R}_q,\tilde{F}_q(\tilde{v}_q,\tilde{R}_q)) = \tilde{R}_{q+1}\quad \text { and }\quad \big (\tilde{v}_q + \tilde{F}_q(\tilde{v}_q,\tilde{R}_q),\tilde{R}_{q+1}\big ) \in D_{ER}(\tilde{F}_{q+1}). \end{aligned}$$

By iteration, we obtain the sequence $(\tilde{v}_p,\tilde{R}_p)_{p\ge q}$,

$$\begin{aligned} \tilde{v}_p(t,x)= \sqrt{\alpha }v_p(\sqrt{\alpha }t,x),\quad \tilde{R}_p(t,x) = \alpha R_p(\sqrt{\alpha }t,x), \end{aligned}$$

so that for $p \rightarrow \infty$

$$\begin{aligned} \tilde{R}_p \xrightarrow {C^0}0,\quad \tilde{v}_p \xrightarrow {C^\beta }\tilde{v} \end{aligned}$$

with $\tilde{v}(t,x) = \sqrt{\alpha }v(\sqrt{\alpha }t,x)$, where *v* denotes the limit of the original sequence $(v_q)_{q \in \mathbb {N}}$. The construction in [3] implies $v(0,x) = 0$, and so $\tilde{v}(0,x) = 0$.

Now let $\tilde{R}_{q+1}$ be a random variable with values in $\big \{R^\alpha _{q+1}, \alpha \in [0,1]\big \}$, where $R^\alpha _{q+1}(t,x):= \alpha R_{q+1}(\sqrt{\alpha }t,x)$. By the previous procedure, in the spirit of Definition 2.2, we obtain a sequence of random variables $\{R^\alpha _{p}\}_{p\ge q}$ and corresponding random variables $\{\tilde{v}_p\}_{p\ge q}$. The latter sequence has values in the class of pointwise solutions to (4.1) and converges pathwise to a limit $\tilde{v}$. Hence, $\mathbb {P}_{\tilde{v}_p} \longrightarrow \mathbb {P}_{\tilde{v}}$ weakly, and the support of $\mathbb {P}_{\tilde{v}}$ is contained in $\{v^\alpha , \alpha \in [0,1]\}$, where $v^\alpha (t,x):= \sqrt{\alpha }v(\alpha t,x)$. Clearly, $\mathbb {P}_{\tilde{v}}$ is a nontrivial stochastic solution to (2.1), if $\mathbb {P}_{\tilde{R}_{q+1}}$ is not chosen to be Dirac.

#### Remark 4.1

Let us explain why we follow [3] instead of other convex integration papers. First, we wanted to present our ideas in the context of the Beltrami scheme. Second, the original solution *v* needs to starts in 0, otherwise we cannot ensure that *v* and $v^\alpha$ have the same initial datum. Thus, we need to follow a scheme leading to compactly supported (or, at least, zero initial datum) solutions. Among the rather short list of works on such Beltrami compact support schemes, [3] appeared to be the most suitable one.

### Towards a Link with Spontaneous Stochasticity

The previous example is artificial, because the stochastic solution $\mathbb {P}_{\tilde{v}}$ is supported only on scalings of the original convex integration solution *v*. It is also not a true example of spontaneous stochasticity (for which we do not have a precise definition) since we chose a very specific sequence of noises, obtained by a perturbed convex integration iteration. Through the iteration, the first noise dictates all further ones and we do not know whether this sequence has any physical relevance. The aim of this subsection is to describe a possible link of our example to spontaneous stochasticity.

As said in Sect. 2.3, for a result called spontaneous stochasticity the noise $\mathcal {R} = (R_q)_{q\in \mathbb {N}_0}$ needs to be general and physically reasonable, not specifically chosen to our needs. We provide the following thoughts, without claiming to solve any rigorous problem.

For every $q \in \mathbb {N}_0$, let $R_q$ be a $L^1([0,T]\times \mathbb {T}^3;\mathbb {R}^{3 \times 3}_{sym})$-valued noise, such that its distribution $\mathbb {P}_{R_q}$ satisfies

$$\begin{aligned} \mathop {\textrm{supp}}\limits \mathbb {P}_{R_q}\subseteq B_{\delta _q}\cap \mathcal {F}_{\lambda _{q}}. \end{aligned}$$

$B_{\delta _q}$ denotes the ball of radius $\delta _q>0$ around 0 in $L^\infty ([0,T]\times \mathbb {T}^3;\mathbb {R}^{3\times 3}_{sym})$, and $\mathcal {F}_{\lambda _q}$ is the subset of the latter space, consisting of elements with all Fourier-coefficients above a number $\lambda _{q}$ equal to zero, and such that the Fourier-coefficients close to size $\lambda _{q+1}$ are dominant, say uniformly in $t\in [0,T]$. Since here we only aim to describe ideas, we do not define $\mathcal {F}_{\lambda _q}$ in detail. $\delta _q$ and $\lambda _q$ are very small and large, respectively, and decaying, respectively increasing in *q*. We have in mind the sequences $\delta _q$ and $\lambda _q$ from convex integration schemes. In addition, the support of $\mathbb {P}_{R_q}$ may additionally be restricted to a regular subspace, e.g. $C^k([0,T]\times \mathbb {T}^3;\mathbb {R}^3)$, $k \in \mathbb {N}\cup \{\infty \}$. Within these constraints, we choose $\mathbb {P}_{R_q}$ as generic as possible, for instance $R_{q}$ of the form

4.11 $$\begin{aligned} R_{q}\left( x,t\right) =\sum _{k\in K_{\lambda _{q+1}}}\sigma _{q}Z_{t} ^{k}e_{k}\left( x\right) , \end{aligned}$$

where $K_{\lambda _{q+1}}$ describes a set of frequencies *k* up to size $\lambda _{q+1}$, $e_{k}$ are the corresponding Beltrami waves and $Z_{k}$ are independent identically distributed stochastic processes, bounded (to satisfy, together with the intensities $\sigma _{q}$, the constraint in $B_{\delta _{q}}$ but spanning a large variability of trajectories) and $\sigma _{q}$ are real numbers tuned to fulfill the requirement of the constraint $\mathcal {F} _{\lambda _{q}}$. The convergence $\delta _q\longrightarrow 0$ implies $\mathbb {P}_{R_q}\longrightarrow \delta _0$ weakly. Natural questions are:

(i) Is there a sequence $(u_q)_{q\in \mathbb {N}}$ of random very weak solutions to the Euler equations corresponding to $\mathcal {R} = (R_q)_{q\in \mathbb {N}_0}$?
(ii) Does a weak limit point $P'$ of $(\mathbb {P}_{u_q})_{q\in \mathbb {N}_0}$ exist such that $P'(\mathcal {S}) = 1$ ($\mathcal {S}$ denotes the set of very weak solutions to the Euler equations)?
(iii) Is $P'$ a nontrivial stochastic solution to the Euler equations, i.e. is $\mathop {\textrm{supp}}\limits P'$ not a singleton?
(iv) Does the full sequence $(\mathbb {P}_{u_q})_{q\in \mathbb {N}_0}$ converge to $P'$?

To us, it seems that with affirmative answers to these questions, $P'$ deserves to be called a spontaneous stochasticity solution (for which, as said before, we do not have a rigorous definition).

These questions have different nature and are all very difficult. Question (i) is a classical question in the theory of 3*D* Euler equations; one way to “solve” it is to shift to the 2D case [22], where the same problem of spontaneous stochasticity may be posed (but in 3*D* it is believed to be more important). Another one could be to shift to the notions of measure-valued (Young measure) solutions [20, Ch.12.3] or dissipative solutions [18, Ch.4.4], which exist globally, but they are not very weak solutions (convex integration provides global solutions of 3D Euler equations but not so generically to be applied to a generic input $R_{q}$). Or, finally, posing the same question for the 3D Navier–Stokes equations, where at least weak global solutions are known to exist.

Question (ii) is a classical question in the framework of random perturbations of deterministic systems without uniqueness [9]. Typically, from a sequence $\left( u_{q}\right) _{q\in \mathbb {N}_{0}}$ satisfying (i) one can try to extract subsequences which converge, and typically this works and the property $P'\left( \mathcal {S}\right) =1$ holds for all limit points. The convergence of the full sequence to a single *P* (question (iv)) is an extremely difficult problem, and positive answers are known only in very few cases, like [1]. For the conceptual model studied in [19], it was also possible to prove such a claim by a clever renormalization procedure. Maybe with the help of the constraint $\mathcal {F}_{\lambda _{q}}$, a renormalization scheme can be developed also for the Euler equations.

Convex integration is related to (iii), the only question we attempt to address here. Let us assume that for the noises considered below a corresponding sequence of random very weak solutions $(u_q)_{q\in \mathbb {N}_0}$ exists such that there is a subsequence $(u_{q_k})_{q_k}$ converging pathwise to a limit *u*, whose distribution

$$\begin{aligned} P' = \lim _k \mathbb {P}_{u_{q_k}} = \mathbb {P}_u \end{aligned}$$

is supported on $\mathcal {S}$. In this framework, we discuss whether there could be a chance to link the example of the previous subsection to question (iii). Assume $R_{q}$ is, for instance, of the form (4.11) above, with high probability restricted to uniformly small ($\delta _q \ll 1$), highly oscillating $(\lambda _q \gg 1)$ matrix-valued fields, such that the convex integration construction of $\{R^\alpha _q, \alpha \in (0,1]\}$ entails that the latter set belongs to $\mathop {\textrm{supp}}\limits \mathbb {P}_{R_q}$. If for large *q*, $\mathop {\textrm{supp}}\limits \mathbb {P}_{R_q}$ is concentrated in a suitable sense, one may find, for some $c_0 >0$,

4.12 $$\begin{aligned} \liminf _q \mathbb {P}_{R_q}\big (\{R^\alpha _q, \alpha \in (0,1]\}\big )\ge c_0. \end{aligned}$$

Of course, this inequality has to be understood in a suitable sense, since strictly, we expect $\mathbb {P}_{R_q}\big (\{R^\alpha _q, \alpha \in (0,1]\}\big ) =0$. For instance, it may be understood as

4.13 $$\begin{aligned} \forall \varepsilon>0: \exists c_0>0 \text { such that }\liminf _q \mathbb {P}_{R_q}\big (\{R^\alpha _q, \alpha \in (0,1]\}_\varepsilon \big )\ge c_0, \end{aligned}$$

where for a set *A* of a metric space (*X*, *d*) we set $A_\varepsilon := \{x: d(x,A)<\varepsilon \}$. We do not specify the metric used to define $\{R^\alpha _q, \alpha \in (0,1]\}_\varepsilon$, maybe the uniform distance on $L^\infty ([0,T]\times \mathbb {T}^3;\mathbb {R}^{3\times 3}_{sym})$ is appropriate.

We may think of $u_q$ as a stochastic process given by a map $\Gamma$, mapping matrices to vector fields,

$$\begin{aligned} u_q = \Gamma \circ R_q. \end{aligned}$$

Therefore, $\mathbb {P}_{u_q} = \mathbb {P}_{R_q}\circ \Gamma ^{-1}$, and (4.12) implies

4.14 $$\begin{aligned} \liminf _q \mathbb {P}_{u_q}\big (\{v^\alpha _q, \alpha \in (0,1]\}\big )\ge c_0, \end{aligned}$$

which should be understood in the same way as (4.12), for instance similarly to (4.13). The hope is now to infer from (4.14) and the pathwise convergence $v_q^\alpha \longrightarrow v^\alpha$ that

$$\begin{aligned} \mathbb {P}_u(v^\alpha , \alpha \in (0,1]) \ge c_0 \end{aligned}$$

(again understood in a suitable sense) which implies that $\mathop {\textrm{supp}}\limits \mathbb {P}_u$ is not a singleton, giving a positive answer to (iii).

As a final remark we point out that the above map $\Gamma$ (assume it exists) is necessarily discontinuous between the topological spaces in which $u_q$ and $R_q$ are considered. Indeed, if $\Gamma$ was continuous, then

$$\begin{aligned} \mathbb {P}_{u_q} \longrightarrow \delta _{\Gamma (0)} \end{aligned}$$

(where 0 denotes the trivial $3\times 3$-matrix) follows from $u_q = \Gamma \circ R_q$ and $\mathbb {P}_{R_q} \longrightarrow \delta _{0}$. In this case, the weak limit of $(\mathbb {P}_{u_q})_{q\in \mathbb {N}_0}$ is a singleton and not a spontaneous stochasticity solution.

Despite not solving any of the questions posed above in a strict sense, we hope that the ideas presented in this subsection may be valuable for some readers and may eventually spark further progress towards a link between a rigorous notion of spontaneous stochasticity and convex integration.

## Data Availability

My research didn’t generate data or I reused existing data.

## Appendix

Here, appealing to [3], we give more (yet not all) details regarding the definition of $F_q$ and $G_q$, introduced in Sect. 4.2. We start with the following lemma, which is standard in convex integration constructions, see for instance Proposition 1.1. and Lemma 1.2. in [3].

### Lemma A.1

(i) Let $\bar{\lambda }\ge 1$, $k \in \mathbb {Z}^3$, $|k|=\bar{\lambda }$, $A_k \in \mathbb {R}^3$ such that
  $$\begin{aligned} A_k \cdot k = 0, \quad |A_k| = \frac{1}{\sqrt{2}},\quad A_{-k} = A_k, \end{aligned}$$
  and set $B_k = A_k + i\frac{k}{|k|} \times A_k$. Let $W^k_{\bar{\lambda }}(x) = B_k e^{i k \cdot x}$, which are called *Beltrami waves*. Then for any choice of $a_k \in \mathbb {C}$ with $\overline{a_k} = a_{-k}$, the vector field
  $$\begin{aligned} W = \sum _{|k| = \bar{\lambda }} a_k W^k_{\bar{\lambda }} \end{aligned}$$
  is real-valued, divergence-free and satisfies
  $$\begin{aligned} \mathop {\textrm{div}}\limits (W\otimes W) = \nabla \frac{|W|}{2}, \end{aligned}$$
  as well as
  $$\begin{aligned} |\mathbb {T}^3|^{-1}\int _{\mathbb {T}^3} W\otimes W dx = \frac{1}{2} \sum _{|k|=\bar{\lambda }} |a_k|^2 \bigg (\mathop {\textrm{Id}}\limits - \frac{k}{|k|}\otimes \frac{k}{|k|}\bigg ). \end{aligned}$$
(ii) There is $r_0>0$, $\bar{\lambda }>1$, symmetric disjoint sets $\Lambda _j \subseteq \{k \in \mathbb {Z}^3: |k| = \bar{\lambda }\}$, $j \in \{1,2\}$, and nonnegative functions $\gamma ^j_k \in C^\infty (B_{r_0}(\mathop {\textrm{Id}}\limits );\mathbb {R})$, $j \in \{1,2\}$ with $\gamma ^j_k = \gamma ^j_{-k}$ such that
  $$\begin{aligned} R = \frac{1}{2} \sum _{k \in \Lambda _j} \bigg (\gamma ^j_k(R)\bigg )^2 \bigg (\mathop {\textrm{Id}}\limits - \frac{k}{|k|}\otimes \frac{k}{|k|}\bigg ),\quad \forall R \in B_{r_0}(\mathop {\textrm{Id}}\limits ), j \in \{1,2\}. \end{aligned}$$

One can choose $\bar{\lambda } = \lambda _{q+1}$ (the latter as in Sect. 4.1), $r_0$ independent from *q*, and in this case we write $W^k_q = W^k_{\lambda _{q+1}}$. For simplicity of notation, we also write $\gamma _k$ instead of $\gamma ^j_k$.

We define $F_q = F_q^1+F_q^2$ as follows. For $(v,R)\in C^1([0,T]\times \mathbb {T}^3;\mathbb {R}^3)\times C^1([0,T]\times \mathbb {T}^3;\mathbb {R}^{3\times 3}_{sym})$, define $a^{kl}_q(v,R)$ by

$$\begin{aligned} a^{kl}_q(v,R)(t,x):= (2r_0^{-1}\big |\big |R(l\mu _q^{-1})\big |\big |_{C^0})^{\frac{1}{2}}\gamma _k\bigg (\frac{r_0R^l(t,x)}{2||R(l\mu _q^{-1})||_{C^0}}\bigg ), \end{aligned}$$

where $R^l$ is the unique matrix-valued solution to the transport equation

A.1 $$\begin{aligned} {\left\{ \begin{array}{ll} \partial _t R^l + v \cdot \nabla R^l = 0, \\ R^l(l\mu _q^{-1},x) = 2r_0^{-1}\big |\big |R(l\mu _q^{-1})\big |\big |_{C^0} \mathop {\textrm{Id}}\limits - R(l\mu _q^{-1},x), \end{array}\right. } \end{aligned}$$

$\gamma _k \in C^\infty (B_{r_0}(\mathop {\textrm{Id}}\limits );\mathbb {R})$ are the functions from Lemma (A.1), $l\in \mathbb {N}$, and $\mu _q \gg 1$ is a suitably chosen parameter. Moreover, $W^k_q$ are the Beltrami waves from Lemma (A.1), and $\Phi ^l$ is the unique vector-valued solution to

A.2 $$\begin{aligned} {\left\{ \begin{array}{ll} \partial _t \Phi ^l + v \cdot \nabla \Phi ^l = 0, \\ \Phi ^l(l\mu _q^{-1},x) = x. \end{array}\right. } \end{aligned}$$

Systems (A.1) and (A.2) are considered on $\mathbb {R}^3$ (to this end, *v* is considered a periodic vector field on $\mathbb {R}^3$). It follows that the solutions to both systems are periodic as well, and hence are considered as maps on $\mathbb {T}^3$. For $(v,R) = (v_q,R_q)$, we write $R^l_q$ and $\Phi ^l_q$ instead of $R^l$ and $\Phi ^l$. Finally, $\chi = \chi _q$ is a non-negative cut-off function with support in $(-\frac{1}{2} - \frac{\lambda _{q+1}^{-\varepsilon _1}}{4},\frac{1}{2} + \frac{\lambda _{q+1}^{-\varepsilon _1}}{4})$ for suitable $\varepsilon _1>0$, and $\chi ^l_q(t):= \chi (\mu _q t - l)$. Define

$$\begin{aligned} F^1_{q}(v,R)(t,x):= \sum _{k,l}\chi ^l_q(t)a_q^{kl}(v,R)(t,x)W^k_q(\Phi ^l(t,x)), \end{aligned}$$

where the summation occurs over the finitely many pairs (*k*, *l*), $l \in \mathbb {N}\cap [0,\lceil T\mu _q\rceil ]$ and $k \in \Lambda _j$, $j \in \{1,2\}$, where $\Lambda _j\subset \mathbb {Z}^3$ are the finite sets from Lemma (A.1). Likewise, define

$$\begin{aligned} F^2_{q}(v,R)(t,x):= &   \sum _{k,l}\chi ^l_q(t)\bigg (\frac{i}{\lambda _{q+1}}\nabla a^{kl}_q(v,R)(t,x)- a^{kl}_q(v,R)(t,x)(D\Phi ^l(t,x)-\mathop {\textrm{Id}}\limits )k)\bigg )\\  &   \times \frac{k}{|k|^2}W^k_q(\Phi ^l(t,x)). \end{aligned}$$

Then $F_q$ from (4.3) is given by $F_{q} = F^1_{q}+F^2_{q}$, while $G_{q}$ is given by

A.3 $$\begin{aligned} G_{q}(v,R,w^1,w^2) =&\,\mathcal {R}\big ((\partial _t + v \cdot \nabla ) w\big ) + \mathcal {R}\big (w\cdot \nabla v\big ) \nonumber \\&+ w^1\otimes w^2 + w^2\otimes w \nonumber \\&+ \sum _l (\chi ^l_q)^2(R^l + R) + \mathcal {R}\mathop {\textrm{div}}\limits \big (w^1\otimes w^2 - \sum _l( \chi ^l_q)^2R^l\big ), \end{aligned}$$

where we set $w:= w^1+w^2$, and denote by $\mathcal {R}$ the right-inverse of the divergence operator, mapping 3*D* vector fields to trace-free $3 \times 3$-matrices, see [3, Lem.1.4]. Again, we omitted spatial mollifications as well as terms arising from trace-free conditions on the appearing matrices. $F_q$ and $G_q$ depend on *q* via $\mu _q$ and $\lambda _{q+1}$, and changing these parameters, in principle, effects the domain and definition of $F_q$ and $G_q$.

## Footnotes

1 We thank Benjamin Favier for pointing out these questions to us.

2 We assume that $\mathcal {S}$ is a Borel set of $L^{2}\left( \left[ 0,T\right] \times \mathbb {T}^{d};\mathbb {R}^d\right)$, otherwise we have to use the external measure.
